# Controlling the spacing of the linked graphene oxide system with dithiol linkers under confinement†

**DOI:** 10.1039/d3na00324h

**Published:** 2023-08-14

**Authors:** Nikita Sugak, Hien Pham, Abhaya Datye, Shomeek Mukhopadhyay, Haiyan Tan, Min Li, Lisa D. Pfefferle

**Affiliations:** a Department of Chemical and Environmental Engineering, Yale University PO Box 208286 New Haven CT 06510-8286 USA nikita.sugak@yale.edu; b Department of Chemical & Biological Engineering, University of New Mexico Albuquerque NM 87131 USA; c CAMMA Laboratory, Institute of Materials Science, University of Connecticut PO Box 06269 Storrs CT USA; d Materials Characterization Core Yale West Campus West Haven CT 06516 USA

## Abstract

2D nanoscale confined systems exhibit behavior that is markedly different from that observed at the macroscale. Confinement can be tuned by controlling the interlayer spacing between confining layers using organic dithiol linkers. Adjusting spacing and selective intercalation have important impacts for catalysis, superconductivity, spin engineering, sodium ion batteries, 2D magnets, optoelectronics, and many other applications. In this study, we report how reaction conditions and organic linkers can be used to create variable, reproducible spacings between graphene oxide to provide confinement systems. We determined the conditions under which the spacing can be variably adjusted by the type of linker used, the concentration of the linker, and the reaction conditions. Employing dithiol linkers of different lengths, such as three (TPDT) and four (QPDT) aromatic rings, we can adjust the spacing between graphene oxide layers under varied reaction conditions. Here, we show that by varying dithiol linker length and using different reaction conditions, we can reproducibly control the spacing between graphene oxide layers from 0.37 nm to over 0.50 nm.

## Abstract 2

Confinement can be tuned by controlling the interlayer spacing between confining layers using organic dithiol linkers of different lengths, such as three (TPDT) and four (QPDT) aromatic rings under varied reaction conditions.

## Graphical abstract

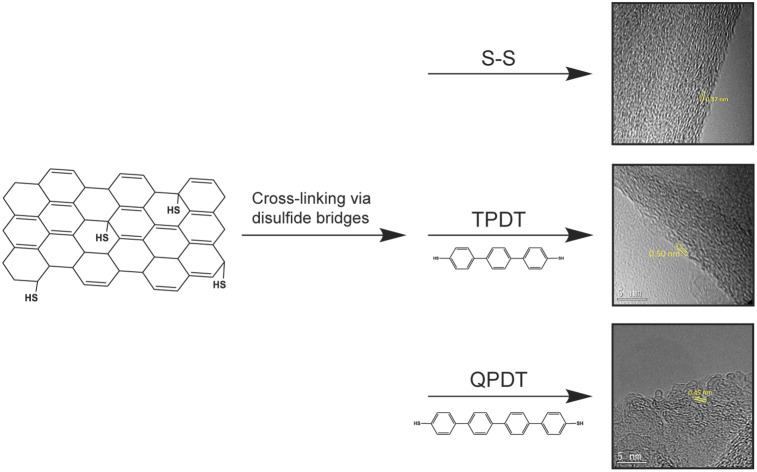

## 1 Introduction

In the past decade, research on nano-confinement using numerous 2D nanomaterials has culminated in remarkable advancements with diverse applications.^1,2^ The confinement geometry and chemical environment can influence an intercalated molecule's conformation modifying, shape, strain, and interaction with the confining material.^3–7^ For instance, it has been demonstrated that confining substances between 2D monolayers and a flat substrate can create internal pressures and unique chemistry at the molecular level.^8^ It has also been observed that confinement affects material properties in different ways. Optical and electronic properties have been changed under the quantum confinement of graphene ribbons.^9^ In a poly(ethylene oxide) (PEO) polymer for optoelectronics, the crystallization behavior of polymers infiltrated into anodized aluminum oxide (AAO) under confinement was investigated and shown to change the crystal orientation varied based on the nanopore size.^10^ Cheng *et al.* discovered changes in mechanical properties that caused the local elastic modulus of PMMA/alumina confined polymer composites to improve by 40%.^11^ The effect of water structures confined between a single layer of graphene and a mica substrate has been reported.^12^ Rubim *et al.* have shown that the physical confinement of graphene oxide (GO) can be achieved as a result of electrostatic interactions between linked layers caused by van der Waals forces. They explored structural transitions and interactions between aqueous lamellar stacks of GO sheets.^13^ The structure of the confined species can be changed by the interactions of the moieties in the confining walls, leading to important tuning of catalytic properties. The main challenge in the study of these systems is that they are quite complex, considering the number of possible interactions involved. It is important to control the spacing between confining layers. Here, we demonstrate how the incorporation of organic linkers can be used to control the interlayer spacing.

The confinement can be adjusted by modification of different oxygen-containing functional groups such as carboxyl, epoxide, and hydroxyl groups, which are located on both sides of the surface of GO.^14–19^ These groups can be used to functionalize and link the GO layers. As an example, linking of the GO layers can be achieved using a broad range of diamine molecules.^20–22^ GO has been cross-lined with diamine monomers including butylenediamine (BDA), *p*-phenylenediamine (PPD), and ethylenediamine (EDA) creating variable interlayer *d*-spacings.^23,24^ Incorporation of other small diamine molecules such as urea was also studied to tune the interlayer *d*-spacings in cross-linked GO. In other studies, Burress *et al.* have shown that the interlayer distances of linked GO with diboronic acid linker increases as the loading of diboronic acid is increased.^25^ Disulfide bridges with various sizes have also been studied to link GO layers.^26^ However, the conditions by which the linker was reacted were not varied. The focus of these past studies was to enhance the properties of cross-linked GO layers for applications in areas such as catalysis, water and gas separation, and desalination. In catalysis, graphene supported VTiO_*x*_ nanoparticles have been used for conversion of methanol to methyl formate^27^ as well as metal free catalysis for oxidative desulfurization.^28^ The versatility of r-GO is that it can promote catalytic reactions by the dislocations, vacancies, edges, impurities, and functional groups present and also by modifying the electronic structure of the encapsulated nanoparticle.^29,30^ All applications in catalysis, energy conversion and storage either use nanoparticles on the r-GO sheets or catalysts encapsulating the nanoparticle complex. Having reproducible control over the spacing between r-GO sheets gives us another control parameter for tuning chemical selectivity for these important reactions.

In the present study, we explored how confinement with varied reproducible spacings in GO can be generated as a function of the reaction conditions under which the linkers are introduced. The confined system is formed by using a dithiol linker to vary the spacing between GO layers. We determined that the spacing can be reproducibly tuned by the linker type and the reaction conditions used to attach the linker. After linking, we characterized the spacing by different techniques, including HRTEM, XPS and XRD analysis. We also explored the changes in phase transitions and spacings in linked GO layers as they are heated, as studied by *in situ* XRD. Spacings are controlled by the type of linker, concentration of linker, and reaction conditions, and the experimental results are repeatable.

## 2 Methods

### 2.1 Synthetic method

GO was purchased from Graphene Supermarket®. As a first step in synthesis, GO was base-washed and then functionalized with thiol functional groups through the formation of epoxide groups on the surface. The synthesis procedures for base-washed and thiol-functionalized graphene oxide were carried out following previous literature.^26,31^

Graphene oxide (500 mg) was mixed in DI water (500 ml) and dispersed in it for 30 min through mild sonication. Then, a solution of 1.25 M NaOH (10 ml) was added and stirred at 70 °C for (1 hour), and centrifuged at 12 500 rpm for 30 min. The samples were collected before re-dispersion in 500 ml of a 0.05 M HCl solution and stirred at 70 °C (1 hour). After the centrifugation, the samples were washed with DI water and filtered. Following filtration, the base-washed GO samples were vacuum dried at room temperature.

Next, the base-washed GO (200 mg) was dispersed in DMSO (50 ml) using mild sonication (1 hour). The solution was placed in an argon-filled glove box, and then potassium thioacetate (20 mg) was added. The mixture was stirred and heated to 50 °C (5.5 hours). After the reaction, the samples were cooled down and centrifuged at 12 500 rpm for 30 min. The samples were collected and washed with acetone (50 ml), diethyl ether (20 ml), and DI water (100 ml) during filtration under vacuum at room temperature. Then, the obtained sample was controllably linked between the layers of GO using one of two dithiol linker molecules, *p*-terphenyl-4,4′-dithiol (TPDT) and [1,1′:4′,1′′:4′′,1′′′-quaterphenyl]-4,4′′′-dithiol (QPDT; Fig. S1†). The disulfide bond formation procedure is described in Section 2.1.1.

**Fig. 1:**
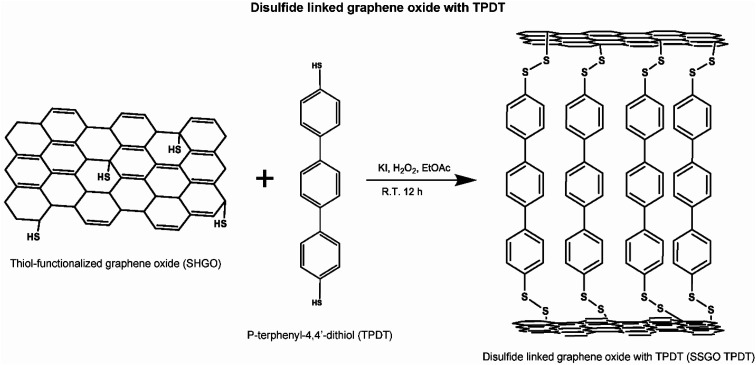
Synthesis scheme.

This process is shown in the figure below (Fig. 1).

TPDT is available commercially, but we synthesized the QPDT. Synthesis of disulfide-linking molecules is reported by Yoo *et al.*^26^ To explore the impact of the structure of a linker component on confinement effect, TPDT and QPDT were used as functional organic linkers in the graphene oxide. The synthesis of [1,1′:4′,1′′:4′′,1′′′-quaterphenyl]-4,4′′-dithiol (QPDT) was reported by Baldea and Frisbie *et al.*^32^

#### 2.1.1. Synthesis of linked GO with different disulfide-linking molecules and various concentrations of H_2_O_2_

A procedure for the formation of disulfide bonds has been reported by Kirihara *et al.*^33^ In our procedure, a total of 50 mg of thiol-functionalized GO (prepared as described above) was dispersed in 15 ml of ethyl acetate (EtOAc) for 10 min using a probe sonicator (60 W). After cooling to room temperature, different amounts of disulfide-linking molecules were added. In addition, a reaction was run without a linker allowing the surface thiols to link together. After adding the appropriate organic linker, the mixture was stirred for 10 minutes. Then, 1.67 mg of KI was added and mixed for an additional 10 minutes, followed by the addition of 0.11 ml of 30% hydrogen peroxide (H_2_O_2_) and agitating for 24 hours at room temperature. The material was filtered, washed with EtOAc and acetone, and then dried under vacuum.

The formation of disulfides has been employed for the oxidation of thiols with different compounds.^34^ However, disulfide transformation necessitates long reaction time and gives low yield. Here, hydrogen peroxide catalyzed by iodide ions in EtOAc at room temperature produces disulfides in a higher yield than other methods for the transformation of thiols to disulfides. Hydrogen peroxide activates iodine which acts as the catalyst for transformation of thiols to disulfides.^33^

For the first set of disulfides linked graphene oxide with TPDT linker (SSGO TPDT) samples we varied the TPDT concentration holding the H_2_O_2_ constant. Here, 0.11 ml of H_2_O_2_ was used with 0, 21.1, 42.2, and 63.3 mg of the TPDT corresponding to SSGO (no linker), SSGO TPDT (1x linker), SSGO TPDT (2x linker), and SSGO TPDT (3x linker), respectively. We also used SSGO without a linker where the thiol functionalized GO surface can be cross linked to form S–S bonds between graphene oxide layers.

Next, we held the linker concentration constant and varied the H_2_O_2_ concentration. For the set of SSGO TPDT (1x linker) samples, we used 21.1 mg of TPDT and the amounts of H_2_O_2_ varied as follows: 0.11, 0.33, and 0.55 ml. For the set of QPDT samples, 53.6 mg of the QPDT was used with 0.11, 0.33, and 0.55 ml of H_2_O_2_.

### 2.2 X-ray diffraction (XRD)

XRD patterns were collected on a Rigaku SmartLab X-ray diffractometer using a Cu Kα source with a beam energy of 8.04 eV (*λ* = 1.5406 Å). Powder samples were mounted on glass slides and data analysis was performed using PDXL-2 Rigaku software.

### 2.3 X-ray photoelectron spectroscopy (XPS)

The XPS spectra were collected using a monochromatic 1486.7 eV Al Kα X-ray source on PHI VersaProbe II X-ray Photoelectron Spectrometer with a 0.47 eV system resolution. The energy scale has been calibrated using Cu 2p_3/2_ (932.67 eV). Powder samples were pressed onto a double-sided scotch tape on a substrate. For region scan, regular beam size of 200 μm in diameter, 23.500 eV pass energy and 0.1000 eV step size were used, and each spectrum was swept multiple times to improve accuracy. The sulfur regions were scanned at least 15 times, while other elements were scanned between 5–10 times depending on abundance. Data analysis was performed with the software package CasaXPS. Atomic concentrations were calculated according to the following equation:1
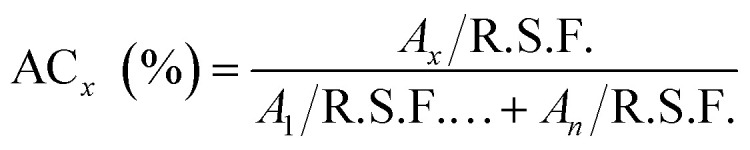
where AC stands for atomic concentration, R.S.F. is relative sensitivity factor, *n* is the total number of different categorized atoms, and *x* is the index for the element of interest.

### 2.4 High-resolution transmission electron microscopy (HRTEM)

All samples were dispersed in ethanol and mounted on holey carbon grids for examination in a JEOL 2010F 200 kV transmission electron microscope. Images were recorded in bright field (BF) mode. To verify the spacing between GO sheets, ten areas were analyzed in each sample resulting in an estimated error of 1.2–1.5%. These sets were then averaged.

### 2.5 *In situ* XRD

To investigate the effect of temperature on the spacing between linked GO, *in situ* temperature dependent XRD was employed using a Rigaku SmartLab X-ray diffractometer using a Cu Kα source (2*θ* = 10–40°, scan rate of 5° min^−1^, step degree of 0.01°) with a beam energy of 8.04 eV (*λ* = 1.5406 Å).

### 2.6 X-ray absorption fine structure (XAFS)

XAFS was performed using S K-edge XANES spectroscopy at beamline 8-BM, on the National Synchrotron Light Source (NSLS-II), at Brookhaven National Laboratory. The Tender Energy X-ray Absorption Spectroscopy (TES) beamline has an energy range of 2–5.5 keV (1–8 keV as designed) in conjunction with Si (111) double crystal monochromator. The sample was pressed into pellets for a higher intensity signal. Kapton tape (Nitto, #P-224, sulfur-free) was used to hold the pellet into the sample holder. After loading the sample, the position was moved to align the sample and focal length of the sample. Sulfur powder was tested as a reference sample. Sulfur K-edge spectra were acquired using an unfocused mode (beam size of 2.5 μm). Energy scans were performed from 2420 to 2900 eV. Calibration was performed with sulfur powder (S8) as standard, assigning a value of 2472.3 eV.^35,36^ The pre-edge background subtraction was carried out by extrapolating the data in the pre-edge region to higher energy with a straight line and the data was normalized by the edge height using the software Athena.^37^

## 3 Experimental results and analysis

The goal of this work was to understand how reaction conditions affect the spacing between the layers during the incorporation of the linker. We varied two main parameters: (1) the concentration of the linker and (2) the concentration of the H_2_O_2_.

We hypothesize that the spacing between the sheets needs to be adjusted to tune the confinement effects. H_2_O_2_ promotes the formation of the active iodine species, which is a catalyst for the coupling reaction.^38,39^ The spacing is a function of both linker concentration and pH as controlled by the concentration of peroxide used in the synthesis.

### 3.1 Varying concentration of the linker *p*-terphenyl-4,4′-dithiol (TPDT)

**Fig. 2:**
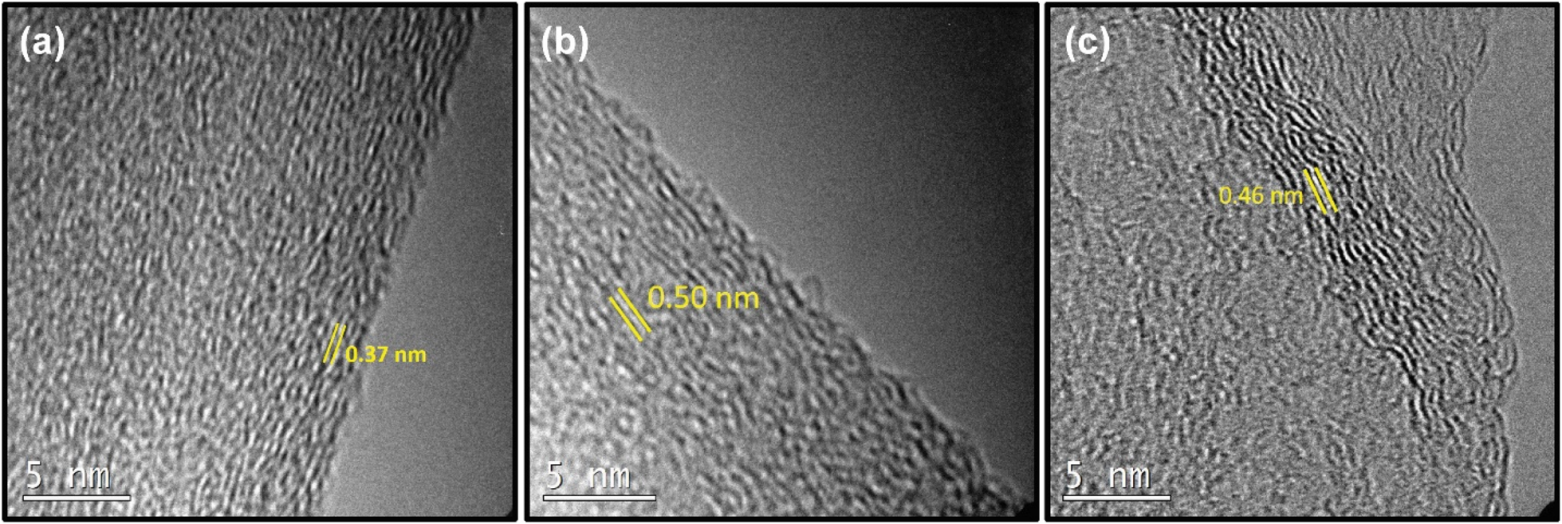
TEM images of (a) SSGO (no linker) (b) SSGO TPDT (1x linker) (c) SSGO TPDT (2x linker).

First, we investigated how spacing between the layers is changed by altering the amount of *p*-terphenyl-4,4′-dithiol (TPDT) used in the synthesis holding other parameters constant. To analyze the effect of the amount of linker on the various spacing between the layers, SSGO (no linker), SSGO TPDT (1x linker), SSGO TPDT (2x linker), and SSGO TPDT (3x linker) were used. The spacing for the TPDT linked GO without the linker (but with S–S bonding) and disulfide GO linked with TPDT are 0.37 nm and 0.50 nm, respectively. HRTEM, XPS, and XRD were used to characterize how the spacing between the sheets varied when using no linker (but coupling of the surface thiol groups), or single, double or triple the amount of TPDT. Fig. 2 shows the HRTEM images of the sample with only S–S linking (SSGO no linker), with the standard TPDT concentration, (1x linker), or double the concentration (2x linker). The spacing corresponds to an average interlayer spacing of 0.37 nm, 0.50 nm, and 0.46 nm, respectively. These results suggest that the higher concentration of linkers causes clustering of the linkers, which leads them to attach to the graphene sheets at an angle. Hence, angling is occurring for the linker with respect to the graphene sheet, potentially explaining the lower spacing. The type of linker, concentration, and reaction conditions all dictate spacing, and results are repeatable. The HRTEM of the three-times linker sample was not performed.

The chemical states of sulfur, oxygen, and carbon for the SSGO (no linker) sample, SSGO TPDT (1x linker), and SSGO TPDT (2x linker) were investigated using high surface sensitivity instrument XPS analysis. We estimated the penetration depth to be 2–10 nm to study the surface structure of the samples on which we detected carbon, oxygen, and sulfur (Table 1).

**Table 1:** Atomic concentration of carbon, oxygen, and sulfur in 1x, 2x, 3x and no linker TPDT samples

Sample	C%	O%	S%
SSGO (no linker)	78%	16%	5%
SSGO-TPDT (1x linker)	80%	13%	7%
SSGO-TPDT (2x linker)	83%	9%	8%
SSGO-TPDT (3x linker)	87%	4%	9%

Table 1 shows the atomic concentration of the surface.

The atomic concentration of sulfur in the SSGO (no linker) sample and the SSGO TPDT (3x linker) sample varies from 5% to 9%. These results suggest that the carbon content increased due to the carbon added from the TPDT linker while surface oxygen was substituted by sulfur. As the concentration of TPDT linker increased from single to double, or double to triple, sulfur content only rose by 1%. SSGO (no linker) and SSGO TPDT (1x linker) were determined to have sulfur atomic concentrations of 5% and 7%, respectively. Assuming that all of the linker was incorporated, this would result in a 2% increase in sulfur from adding the TPDT linker into SSGO TPDT (1x linker). Based on this assumption, we anticipated the sulfur content to be 9.8% and 13.72% in SSGO TPDT (2x linker) and SSGO TPDT (3x linker), respectively. However, the results of S 2p analysis showed lower quantities for 2x and 3x linkers. This may indicate that the linker was not fully incorporated for the higher loadings due to possible clustering of the linkers as the concentration of linkers increases, suggesting that a better swelling agent may improve incorporation.

**Fig. 3:**
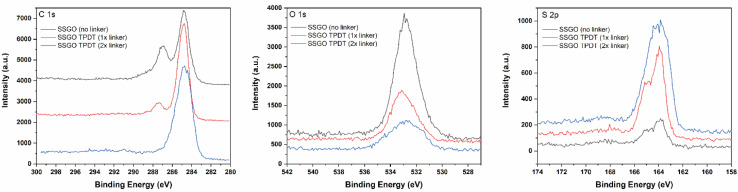
XPS region spectra for SSGO (no linker), SSGO TPDT (1x linker), and SSGO TPDT (2x linker). Carbon 1s region (Left). Oxygen 1s region (Middle). Sulfur 2p region (Right).

These results imply that the linking reaction is proceeding swiftly, and that the linker is bonded between graphene layers at an angle instead of in a straight line perpendicular to another layer of graphene. The S 2p region (Fig. 3) shows a broad peak from 163.3 to 165.1 eV in the case of no linker, 1x, and 2x linker samples and corresponds to thiophene or thiol.^31,40,41^

However, this region overlaps with the doublet peak at 163–164 eV for disulfide (S–S).^26,42–44^ From all of these studies, we conclude that peak fitting is not an accurate method for confirming disulfide bonds since thiol and disulfide bonds overlap significantly. In addition, the XPS S 2p region spectra for thiol functionalized GO (Fig. S2†) display identical S 2p characteristics as SSGO (no linker) and SSGO TPDT (1x linker). Due to this challenge, we used X-ray absorption fine structure (XAFS) spectroscopy to identify the bonding of sulfur species in linked SSGO samples (see Section 3.2). The samples all showed analogous XPS spectra in O 1s, indicating main peaks at 533.1 eV were attributed to C–OH.^45,46^ The O 1s spectra of SSGO with no linker, 1x, and 2x show no significant differences in peak positions, which means that disulfide bonds and linking formation had insignificant impact on the oxygen functional groups other than a decrease in surface oxygen. The C 1s spectrum displays that the main peak at 284.7 eV corresponds to C–C bonds, which is shown in all our materials. A shoulder at 287.3 eV was attributed to carbonyls remaining from the GO.^46,47^ The deconvolution results of the C 1s and O 1s are shown in Fig. S3.†

**Fig. 4:**
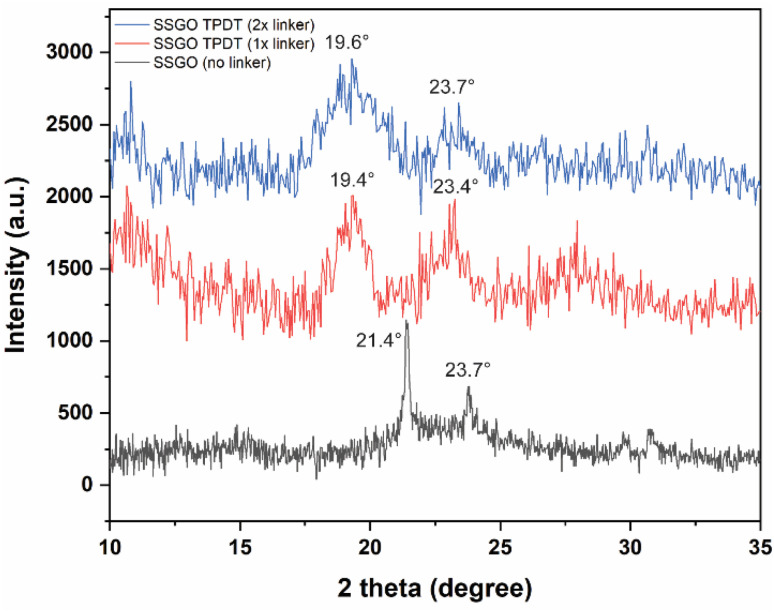
XRD patterns of the SSGO TPDT with no linker, 1x, and 2x the amount of linker.

Fig. 4 displays the XRD patterns of the SSGO TPDT with no linker, 1x, and 2x amounts of the linker. SSGO (no linker) exhibited two main peak positions of SSGO at 21.4° and 23.7°, SSGO TPDT (1x linker) displayed two main peaks at 19.4° and 23.4°, and SSGO TPDT (2x linker) at 19.6° and 23.7°. These results are corroborated by our HRTEM and XPS analyses, which show that the linkers could be attached not perpendicularly but at an angle due to the restacking between the layers and creating blockage for the other linkers. As a result, greater gap distances were observed for the SSGO TPDT 1x linker than for the 2x linker. The diffraction patterns at 23.4° and 23.7° are attributed to disulfide bonds are formed between thiol functionalized GO layers in all linked samples.^26^ However, the peak at 23.7° slightly shifted due to the possible restacking between GO layers. The diffraction peaks of the SSGO TPDT (3x linker) were detected at 18.9° and 23.7° (Fig. S4†). Due to the problems with overlapping of disulfide and thiol peak positions in XPS, we have carried out sulfur K-edge XAFS.

### 3.2 XAS K-edge of linked GO

**Fig. 5:**
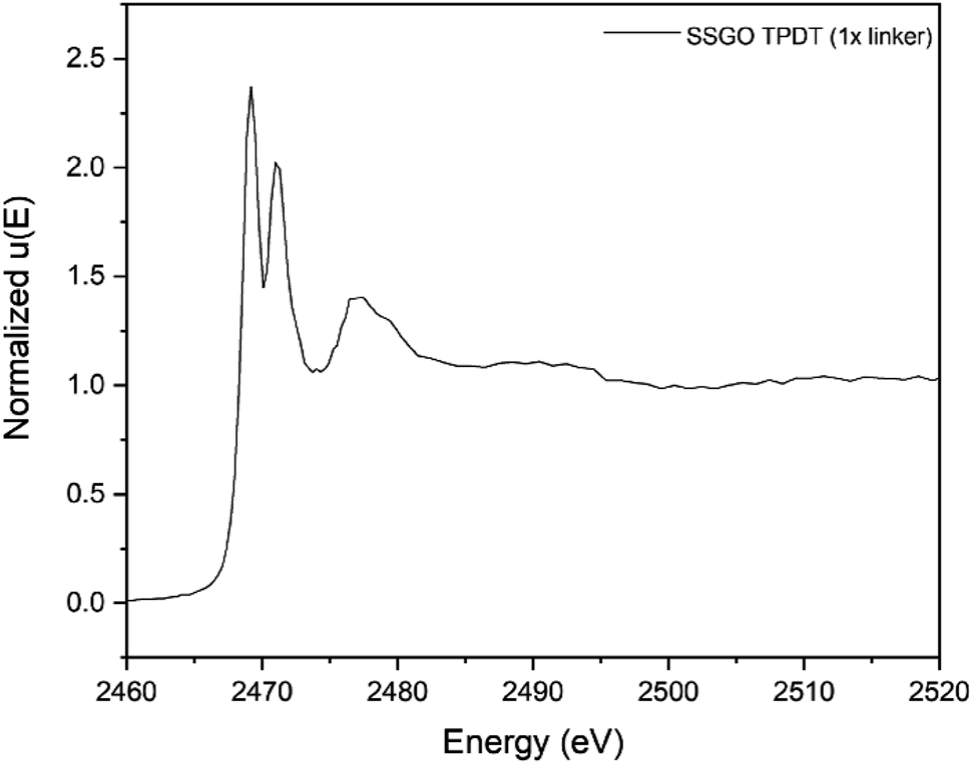
Sulfur K-edge X-ray absorption near-edge spectra of SSGO TPDT (1x linker). The spectra was pre-edge subtracted and normalized.

The capability of XANES to distinguish the presence of sulfur species at different oxidation states is an effective method to identify the bonding of sulfur species in linked SSGO samples. Fig. 5 shows the sulfur K-edge spectra of SSGO TPDT (1x linker). The spectra exhibit three major peaks at 2469.5, 2471.3 and 2478 eV corresponding to the sulfide (S^2−^), disulfide (S–S),^48^ and sulfite (S^4+^).^49^ The spectra show a shoulder at 2478 eV corresponding to sulfites S^4+^. This formation of S–O bonds suggests that some of the sulfur groups are interacting and anchoring at oxygen defect sites in the GO lattice.

Prior studies reported that the disulfide bonds occur at 2469.1 eV with a double peak structure for several compounds such as diphenyl disulfide and dibenzyl disulfide.^50^ Other reports showed similar double peak structure of disulfides using sulfur K-edge X-ray absorption.^48,51,52^ This double peak structure makes it possible to distinguish thiol and disulfide bonds since they appear at the same energies but have single and double peak structures, respectively. Prange *et al.* have shown clear differences in peak shapes between disulfide cystine and thiols cysteine.^36^ Our results indicate the formation of disulfide bonds in the SSGO TPDT (1x linker) sample.

### 3.3 Changing peroxide concentration

**Fig. 6:**
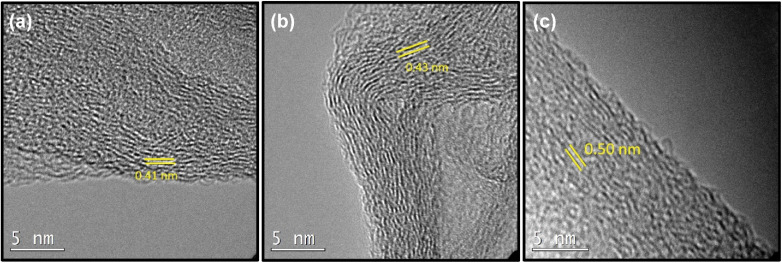
TEM images of (a) SSGO TPDT 0.55 ml of H_2_O_2_ (b) SSGO TPDT 0.33 ml of H_2_O_2_ (c) SSGO TPDT 0.11 ml of H_2_O_2_.

The approach of synthesizing SSGO TPDT is dependent on various concentrations of peroxide that lead to changes in critical parameters such as spacing and pH. According to a previous study, hydrogen peroxide transforms thiols into disulfides depending on the reaction duration and pH.^53,54^ Due to the insolubility of thiols in most organic solvents and their low yield, iodine ions are necessary to convert thiols into disulfides utilizing peroxide as a co-catalyst.^55^ In addition, we chose to adjust the amount of H_2_O_2_ due to its swelling effect. HRTEM was used to examine the morphologies of the distinct SSGO TPDT samples, as illustrated in Fig. 6.

For samples where we used 21.2 mg of TPDT linker, we tested how varying the H_2_O_2_ concentration would affect the spacing. Here 0.55, 0.33, and 0.11 ml of H_2_O_2_ was used; we observed that the spacing became wider as the amount of peroxide is decreased corresponding to 0.41, 0.43, and 0.50 nm, respectively.

Hydrogen peroxide is commonly used to catalyze thiols into disulfides. In the presence of an excessive amount of oxidant, subsequent oxidation of the produced disulfide may also proceed. It has additionally been reported that successful conversion of thiols to disulfides occurs using 1.0 equivalent of 30% hydrogen peroxide in the presence of 1 mol% of catalyst (iodide ions). Therefore, due to the quicker oxidation of iodide ions, the reaction rate increases as the amount of peroxide is increased while the interlayer spacing is decreased. All experiments were carried out several times under the exact condition and showed consistent results with respect to spacing in the HRTEM, XRD and XPS analysis.

**Fig. 7:**
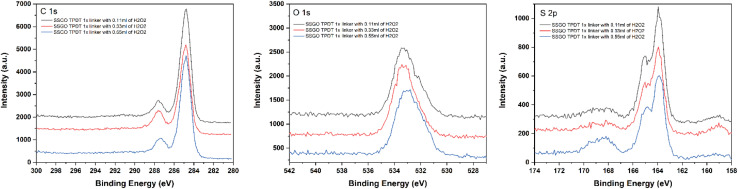
XPS region spectra for SSGO TPDT 1x linker with 0.11, 0.33, and 0.55 ml of H_2_O_2_. Carbon 1s region (Left). Oxygen 1s region (Middle). Sulfur 2p region (Right).

We conducted XPS to determine the effect of peroxide on the surface species on the GO. All samples exhibited similar XPS spectra in the C 1s region (Fig. 7 Left), with the main peak at 284.7 eV corresponding to C–C bonds, as shown in all our H_2_O_2_ samples. The amount of H_2_O_2_ increased but did not noticeably change the shoulder at 287.3 eV. This means that the chemical states of the linked graphene oxide or the carbon functional groups that were not oxidized were barely affected by peroxide. The O 1s spectra of SSGO TPDT with 0.11, 0.33, and 0.55 ml of H_2_O_2_ show one main peak at 533.1 eV that is attributed to C–OH groups. A slight shift in the peak position of SSGO TPDT (0.33 ml) and SSGO TPDT (0.55 ml) to a lower binding energy confirmed the partial oxidation of the carbon-containing functional groups on the surface of GO. The deconvolution results are shown in Fig. S5.†Fig. 7 (right) demonstrates the analogous S 2p spectra of SSGO TPDT (0.11 ml) and SSGO TPDT (0.33 ml). The S 2p region of the SSGO TPDT (0.55 ml) sample contains a new broad peak from 167–170 eV corresponding to oxidized sulfate species due to the excessive amount of H_2_O_2_. It is clear that oxidized sulfur species appear as a result of oxidation of thiol groups. We hypothesize that the high concentration of peroxide causes thiol oxidation, which inhibits the linking reaction. However, the sulfur content in this batch of samples remains constant at 7%, as shown in Table 2. Additionally, Raman spectroscopy and thermogravimetric analysis (TGA) have been used to study the degree of disorder and stability of linked graphene oxide (Fig. S6–S8†).

**Table 2:** Atomic concentration of carbon, oxygen, and sulfur in 0.11, 0.33 and 0.55 ml of peroxide in 1x linker TPDT samples

Sample	C%	O%	S%
SSGO-TPDT (0.11 ml)	80	13	7
SSGO-TPDT (0.33 ml)	77	16	7
SSGO-TPDT (0.55 ml)	77	16	7

**Fig. 8:**
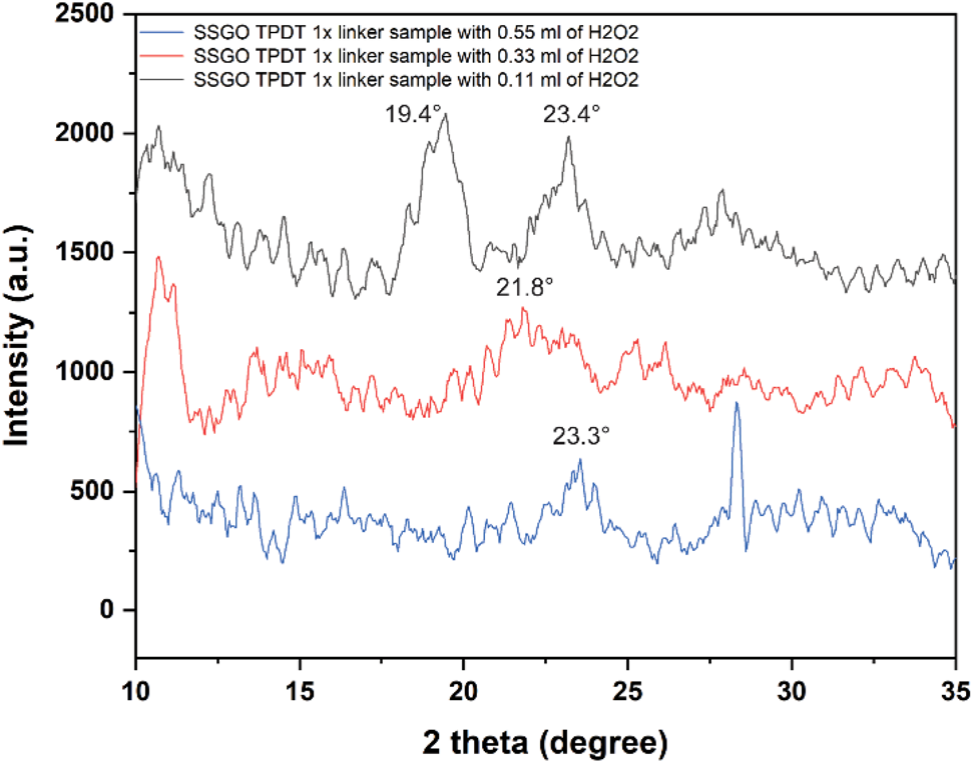
XRD patterns from 10° to 35° for the SSGO TPDT 1x linker with 0.11, 0.33, and 0.55 ml of H_2_O_2_. Raw data is smoothed, and the number of windows was set as 10.

Fig. 8 displays the XRD patterns of the SSGO TPDT sample with different concentrations of peroxide. The broad peak positions of SSGO TPDT (0.11 ml), SSGO TPDT (0.33 ml), and SSGO TPDT (0.55 ml) emerged at 19.4°, 21.8°, and 23.4°, respectively. These findings were consistent with HRTEM analysis, which revealed that as peroxide concentration increased, average interlayer distances shrank.

### 3.4 Varying the size and structure of the linker

Varying the size and structure of the linker is not straightforward. Using QPDT with the same synthesis methodology did not increase the spacing (Fig. S9†). XPS results of SSGO QPDT samples with 0.11, 0.33, and 0.55 ml of H_2_O_2_ are shown in the Table S1 and Fig. S10.† Different swelling agents and synthesis techniques need to be developed to assure that the larger linkers can be transported into the GO layers effectively and the reactive ends linked with the GO. We hypothesize that incorporation of a larger linker such as QPDT is impeded due to the limited volume between linked graphene oxide sheets causing transport constraints.

### 3.5 Change in spacing as the synthesized linked GO is heated

*In situ* temperature-programmed XRD technique can ascertain changes in phase transitions and determine the changes in the interlayer spacing. Both of these variables can be observed by the shift of the peak position in response to thermal treatments at the precise temperature or conditions of interest. Pan *et al.* have shown that the temperature-dependent reduction of GO to graphene occurs with the release of confined water when the *in situ* XRD of GO was explored under different environments.^56^ For instance, it has been discovered that interlayer spacing of GO steadily decreases as temperature increases in the range from 50 °C to 800 °C.^57^ Using Bragg's law, it is known that the position of peaks from an XRD pattern can be correlated to the *d*-spacing between the different crystal planes in a GO sample; therefore, the shift in position of specific peaks in the XRD pattern of GO can be correlated to changes in the average interlayer spacing between adjacent carbon planes. One research group observed that when the GO was exposed in air at 60 °C and 200 °C, the spacing decreased from 7.4 Å to 6.5 Å.^58^ We use *in situ* XRD to investigate the interlayer gap between GO sheets under vacuum as the temperature changes.

**Fig. 9:**
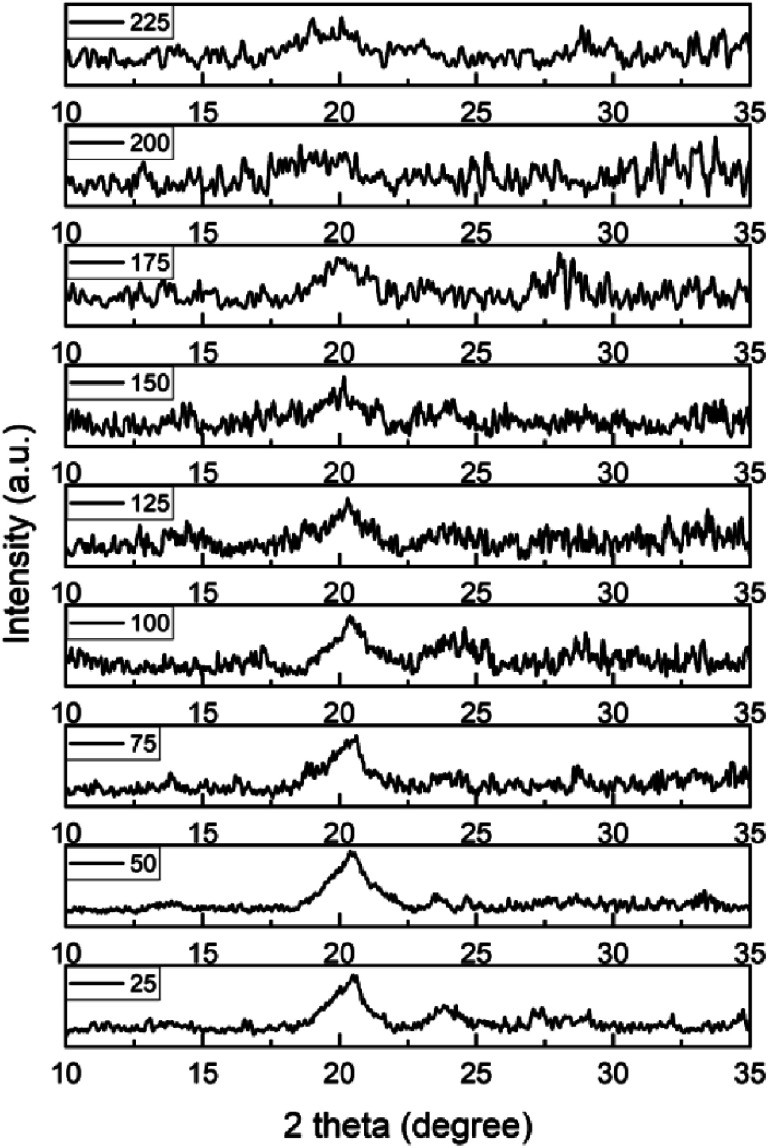
Shift of XRD pattern of a SSGO TPDT (1x linker) with 0.11 ml of H_2_O_2_ in the temperature range between 50 °C and 225 °C.

*In situ* XRD analysis of a SSGO-TPDT (1x linker) with 0.11 ml of H_2_O_2_ sample under different temperatures was performed (Fig. 9). The sample was heated to nine different temperatures in vacuum to examine the impact of thermal reduction: 25 °C, 50 °C, 75 °C, 100 °C, 125 °C, 150 °C, 175 °C, 200 °C, and 225 °C. At room temperature, this sample's *d*-spacing measures 0.50 nm. When sample was heated from 50 °C to 175 °C, two peaks were observed at around 20.4° and 23.4° and the *d*-spacing remained unaffected. Beyond 175 °C, we observed that the peak at 20.4° degraded into a broad peak due to the removal of oxygen species from the surface at temperatures ranging from 200–225 °C.^56^ Consequently, this analysis verifies that we can maintain the interlayer distance using thermal treatments in the tested temperature range, giving us another variable to control the effective pressure for molecules intercalated between the GO layers. Yu *et al.* investigated the sulfur stability of graphene and GO, demonstrating that sulfur enhances thermal stability when thiol–ene groups are introduced.^59^ We observe that sulfur incorporation facilitates retaining spacing during thermal treatment.

## 4. Conclusions

We have investigated the use of dithiol linkers to control the spacing of GO under different reaction conditions to provide a confinement system. The spacing is affected by synthesis parameters in a reproducible manner. Using a larger organic linker than TPDT causes transport issues to interfere with adjustment of interlayer spacings in the GO. Different swelling agents are likely necessary to make use of a QPDT linker. We found that as the concentration of H_2_O_2_ is increased, the spacing decreases consistently. A higher concentration of TPDT linkers decreases the interlayer spacing, possibly due to the clustering of the linkers and their formation at different angles. Adjusting the spacing and selective intercalation of different molecules between linked graphene oxide under confinement has significant potential to improve and tune the properties of optoelectronics, 2D magnets, membranes for separation, catalysts, and many other applications.

## Author contributions

NS is the corresponding author; conceptualization, NS, LDP; methodology, NS, HP, AD, SM, HT, ML, LDP; investigation, NS, HP, LDP; writing – original draft, NS, LDP; writing – review & editing, all authors; project administration, LDP; funding acquisition, LDP.

## Conflicts of interest

There are no conflicts to declare.

## Supplementary Material

NA-005-D3NA00324H-s001
