# Supplementary material for: Controlling the spacing of the linked graphene oxide system with dithiol linkers under confinement
Source: Nanoscale Adv. 2023 Aug 14;5(17):4553–62. doi: 10.1039/d3na00324h (PMC10448350; doi:10.1039/d3na00324h)
Supplement: NA-005-D3NA00324H-s001 [file NA-005-D3NA00324H-s001.pdf]

Electronic Supplementary Material (ESI) for Nanoscale Advances.
This journal is © The Royal Society of Chemistry 2023

Supplementary information

Controlling the spacing of the linked graphene oxide system with dithiol linkers under confinement

Nikita Sugak[a]*, Hien Pham[b], Abhaya Datye[b], Shomeek Mukhopadhyay[a], Haiyan Tan[c], Min Li[d], Lisa D. Pfefferle[a]

[a] Department of Chemical and Environmental Engineering, Yale University, PO Box 208286, New Haven, CT, USA

[b] Department of Chemical & Biological Engineering, University of New Mexico, Albuquerque NM 87131, USA

[c] CAMMA Laboratory, Institute of Materials Science, University of Connecticut, PO Box 06269, Storrs, CT, USA

[d] Materials Characterization Core, Yale West Campus, West Haven, CT 06516, USA

Corresponding Author: Nikita Sugak

PO Box 208286, New Haven CT 06510-8286, USA

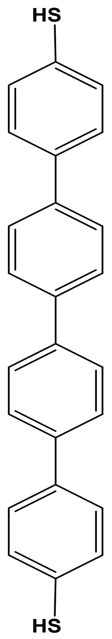

[1,1':4',1'':4'',1'''-quaterphenyl]-4,4''-dithiol (QPDT)

Figure S1. Structure of QPDT.

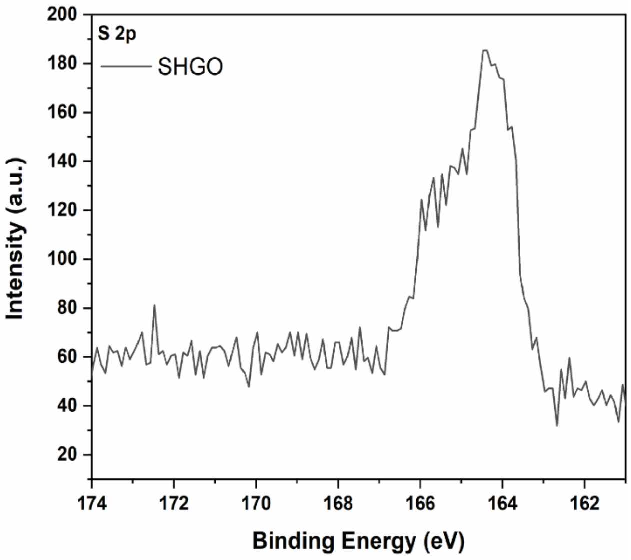

Figure S2. XPS S2p region spectra for SHGO sample.

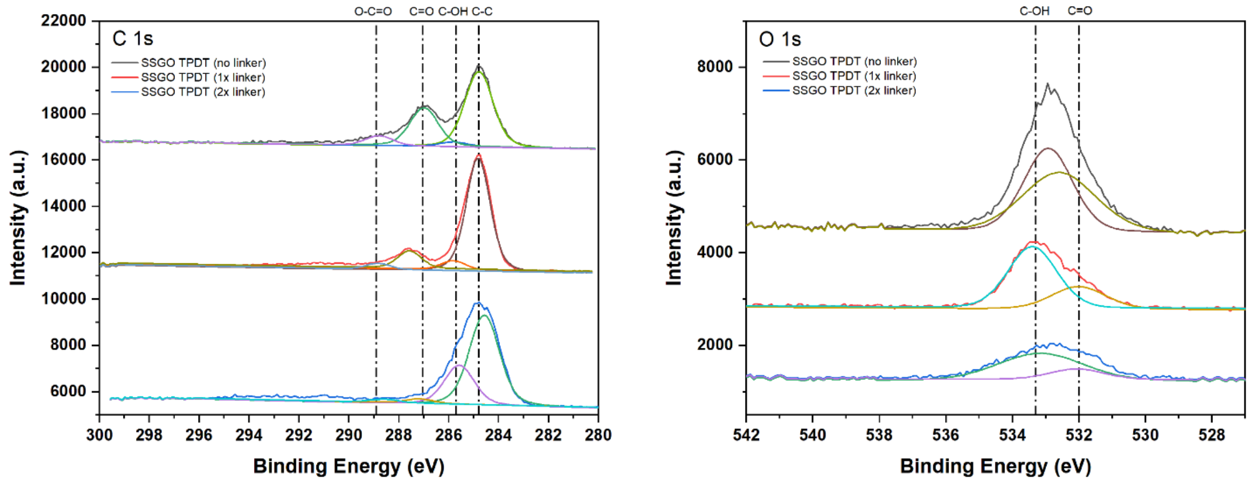

Figure S3. Deconvolution results of the XPS spectra for SSGO (no linker), SSGO TPDT (1x linker), and SSGO TPDT (2x linker). Carbon 1s region (**Left**). Oxygen 1S region (**Right**).

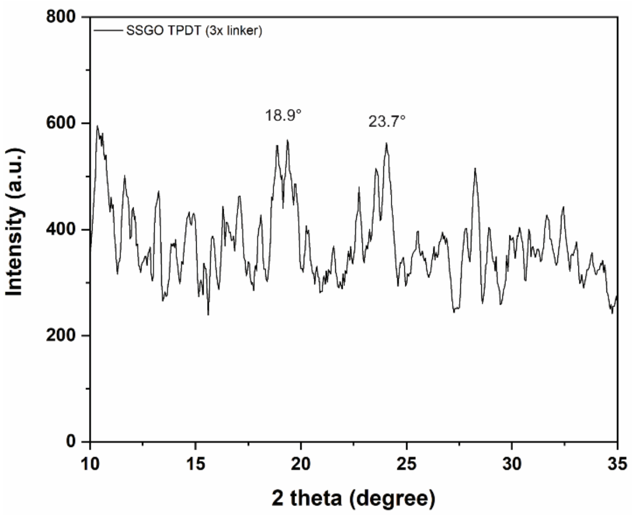

Figure S4. XRD Pattern of the SSGO TPDT 3x linker sample. The diffraction pattern of the SSGO TPDT (3x linker) were detected at 18.9˚ and 23.7˚.

Table S1 – Atomic concentration of carbon, oxygen, and sulfur in 0.11, 0.33 and 0.55 ml of peroxide in 1x times QPDT samples.

Sample	C%	O%	S%
SSGO-QPDT (0.11 ml)	75%	23%	2%
SSGO-QPDT (0.33 ml)	78%	20%	2%
SSGO-QPDT (0.55 ml)	79%	18%	3%

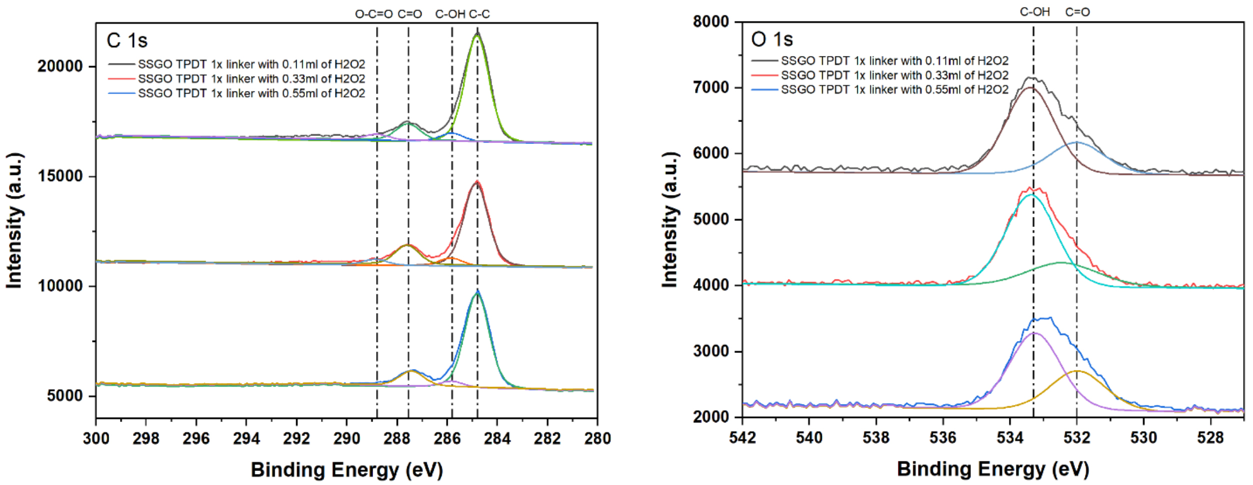

Figure S5. Deconvolution results of the XPS spectra for SSGO TPDT 1x linker with 0.11, 0.33, and 0.55 ml of H_2O_2. Carbon 1s region (**Left**). Oxygen 1S region (**Right**).

Raman spectroscopy was performed on a HORIBA LabRAM HR Evolution Raman microscope with a 532 nm laser.

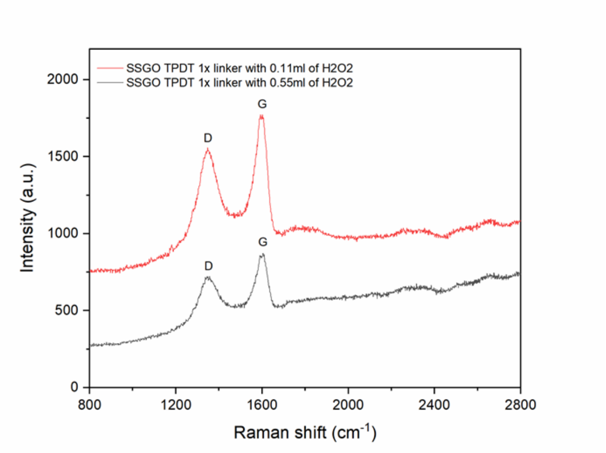

Figure S6. Raman spectra for SSGO TPDT 1x linker with 0.11 and 0.55 ml of H_2O_2.

Figure S6 shows the Raman spectrum of SSGO TPDT with 0.11 ml and 0.55 ml of H_2O_2. The Raman spectra displays D and G peaks located at 1344 and 1592cm^{-1}, respectively. The intensity ratios of I_D/I_G of SSGO TPDT with 0.11 ml and 0.55 ml of H_2O_2 are 0.84 and 0.83, respectively.

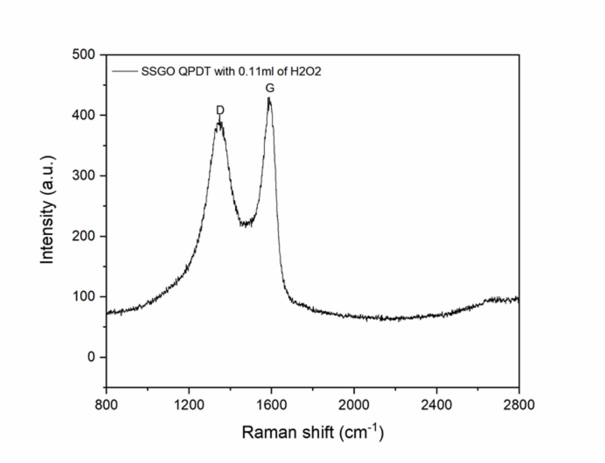

Figure S7. Raman spectra for SSGO QPDT with 0.11 ml of H_2O_2.

Figure S7 shows the Raman spectra of SSGO QPDT with 0.11 ml of H_2O_2. The Raman spectra indicates D and G peaks found at 1346 and 1593cm^{-1}, respectively. The intensity ratio of I_D/I_G of SSGO QPDT with 0.11 ml of H_2O_2 is 0.91.

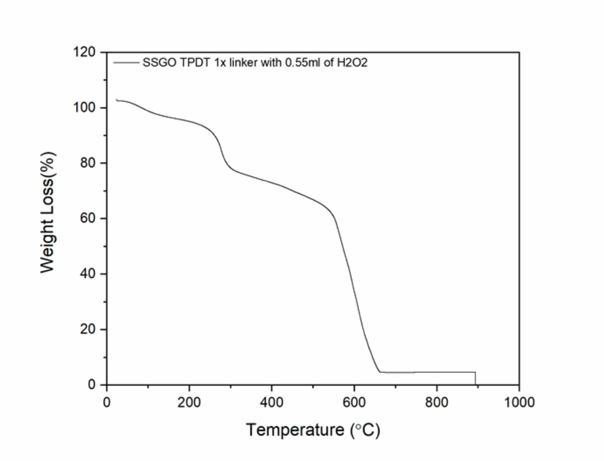

Figure S8. TGA weight loss curve for SSGO TPDT with 0.55 ml of H_2O_2.

The investigations of the thermal stability of the SSGO TPDT 1x linker with 0.55 ml of H_2O_2 were performed using TGA. TGA was recorded on a TGA Q50 V20.13 system at the heating rate of 20K/min from 25-900 °C under air. The thermogravimetric curve showed two major weight drops. The first effect observed between the temperatures of 100 and 300 °C is associated with the thermal removal of oxygen functionalities (the decomposition of carboxylic) chemically bonded with graphene layers and the release of CO_2 gas. The second significant loss of sample weight begins at the temperature of around 550 °C. This effect corresponds to the decomposition of graphene-like sheets.

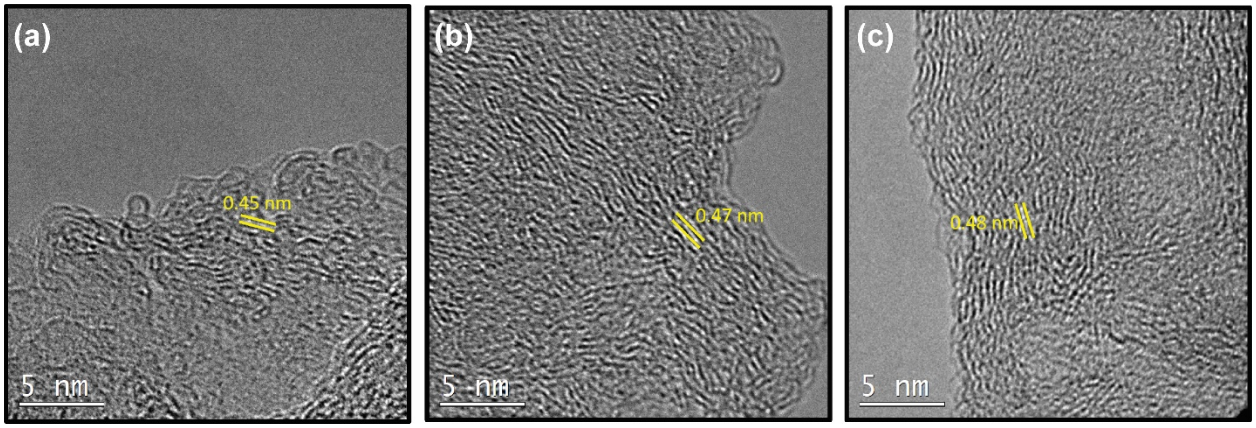

Figure S9. TEM images of a) SSGO QPDT (1x linker) with 0.11 ml of H_2O_2 b) SSGO QPDT (1x linker) with 0.33 ml of H_2O_2 c) SSGO QPDT (1x linker) with 0.55 ml of H_2O_2

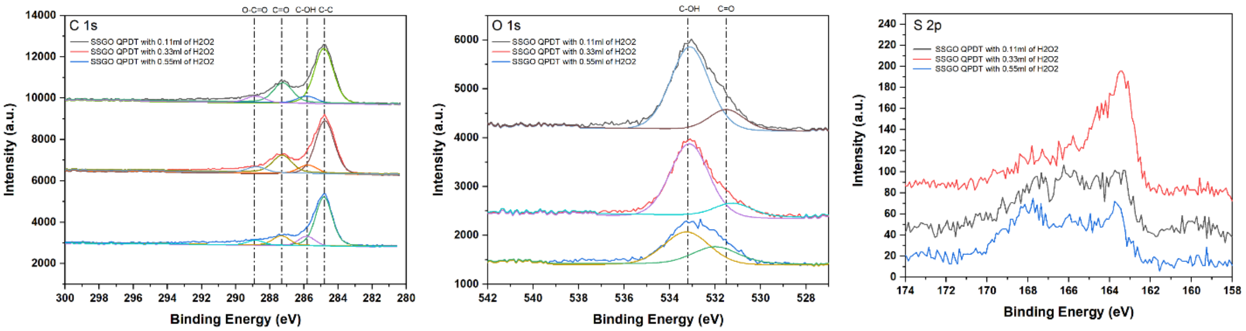

Figure S10. Deconvolution results of the XPS spectra for SSGO QPDT samples with 0.11, 0.33, and 0.55 ml of H_2O_2. Carbon 1s region (**Left**). Oxygen 1S region (**Middle**). Sulfur 2p region (**Right**).
